# The COVID-19 Citizen Science Study: Protocol for a Longitudinal Digital Health Cohort Study

**DOI:** 10.2196/28169

**Published:** 2021-08-30

**Authors:** Alexis L Beatty, Noah D Peyser, Xochitl E Butcher, Thomas W Carton, Jeffrey E Olgin, Mark J Pletcher, Gregory M Marcus

**Affiliations:** 1 Division of Cardiology University of California, San Francisco San Francisco, CA United States; 2 Louisiana Public Health Institute New Orleans, LA United States; 3 Department of Epidemiology and Biostatistics University of California, San Francisco San Francisco, CA United States

**Keywords:** COVID-19, digital technology, participant engagement, electronic health records, mobile app, mHealth, digital health

## Abstract

**Background:**

The COVID-19 pandemic has catalyzed a global public response and innovation in clinical study methods.

**Objective:**

The COVID-19 Citizen Science study was designed to generate knowledge about participant-reported COVID-19 symptoms, behaviors, and disease occurrence.

**Methods:**

COVID-19 Citizen Science is a longitudinal cohort study launched on March 26, 2020, on the Eureka Research Platform. This study illustrates important advances in digital clinical studies, including entirely digital study participation, targeted recruitment strategies, electronic consent, recurrent and time-updated assessments, integration with smartphone-based measurements, analytics for recruitment and engagement, connection with partner studies, novel engagement strategies such as participant-proposed questions, and feedback in the form of real-time results to participants.

**Results:**

As of February 2021, the study has enrolled over 50,000 participants. Study enrollment and participation are ongoing. Over the lifetime of the study, an average of 59% of participants have completed at least one survey in the past 4 weeks.

**Conclusions:**

Insights about COVID-19 symptoms, behaviors, and disease occurrence can be drawn through digital clinical studies. Continued innovation in digital clinical study methods represents the future of clinical research.

**International Registered Report Identifier (IRRID):**

DERR1-10.2196/28169

## Introduction

The ubiquity of the internet, computers, and smartphones has enabled new ways for people to participate in clinical studies [1]. Traditional research methods involve direct interaction between research staff and participants with laborious and costly efforts to recruit, enroll, consent, and follow participants [2]. The creation of digital tools for conducting clinical studies has made it possible to engage large numbers of people in research studies, collect data beyond the clinical setting, avoid constraints related to geographic location or proximity to a research center, collect frequent participant-reported outcomes, collect data from connected devices such as smartphones and wearables, and rapidly generate results to inform advances in health and science [1,3].

In 2014, University of California, San Francisco (UCSF) researchers launched the Health eHeart Study [4], a ground-breaking cohort study conducted using web- and mobile-based recruitment, enrollment, consent, and participation [5-8]. Recognizing the potential of this approach to facilitate clinical research more broadly, the infrastructure of the Health eHeart Study was expanded to develop the NIH-supported Eureka Research Platform [9] for developing and hosting digital clinical studies [10-12]. The platform enables studies to be conducted entirely through web and mobile interactions, as well as studies with digital interaction as a complement to traditional clinical research methods. The Eureka Research Platform has hosted over 40 studies.

The COVID-19 pandemic has catalyzed innovation in clinical investigation and especially digital methods for clinical studies [3]. The risk of virus transmission has created a need to limit face-to-face interactions between study participants and research staff, highlighting the need for digital approaches [13]. Despite the challenges, researchers have rapidly innovated to solve an urgent global health crisis, and citizens of the world have been inspired to contribute to research studies in unprecedented numbers [3,14-17].

Here, we describe the methods of the COVID-19 Citizen Science (CCS) study, an entirely digital clinical study on the Eureka Research Platform. The objectives of the study are to generate knowledge about participant-reported COVID-19 symptoms, behaviors, and disease occurrence to facilitate the public health response to COVID-19. These methods highlight clinical study innovations and future directions for advancing science using digital clinical study methods.

## Methods

### Study Design, Setting, and Participants

**Figure 1:**
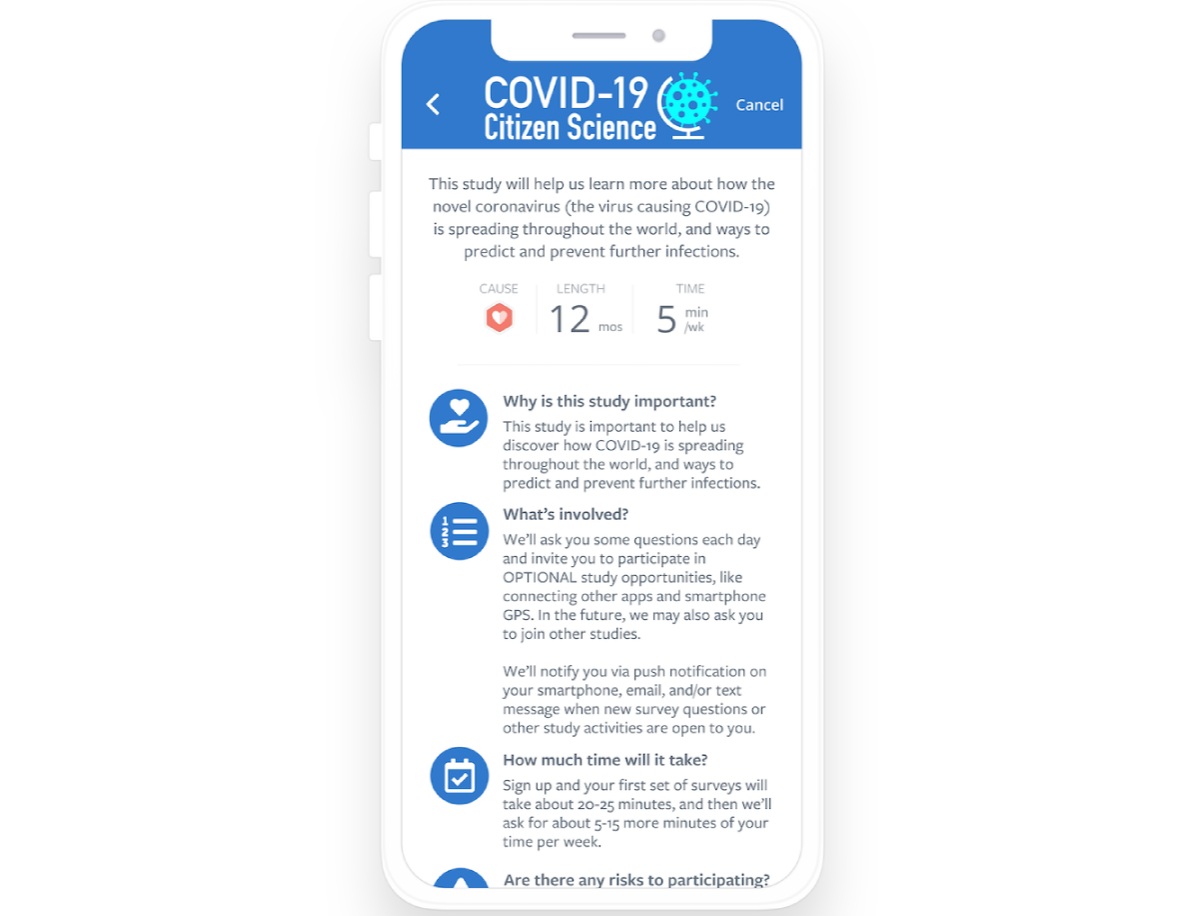
Screenshot of the COVID-19 Citizen Science study app on the Eureka Research Platform.

**Figure 2:**
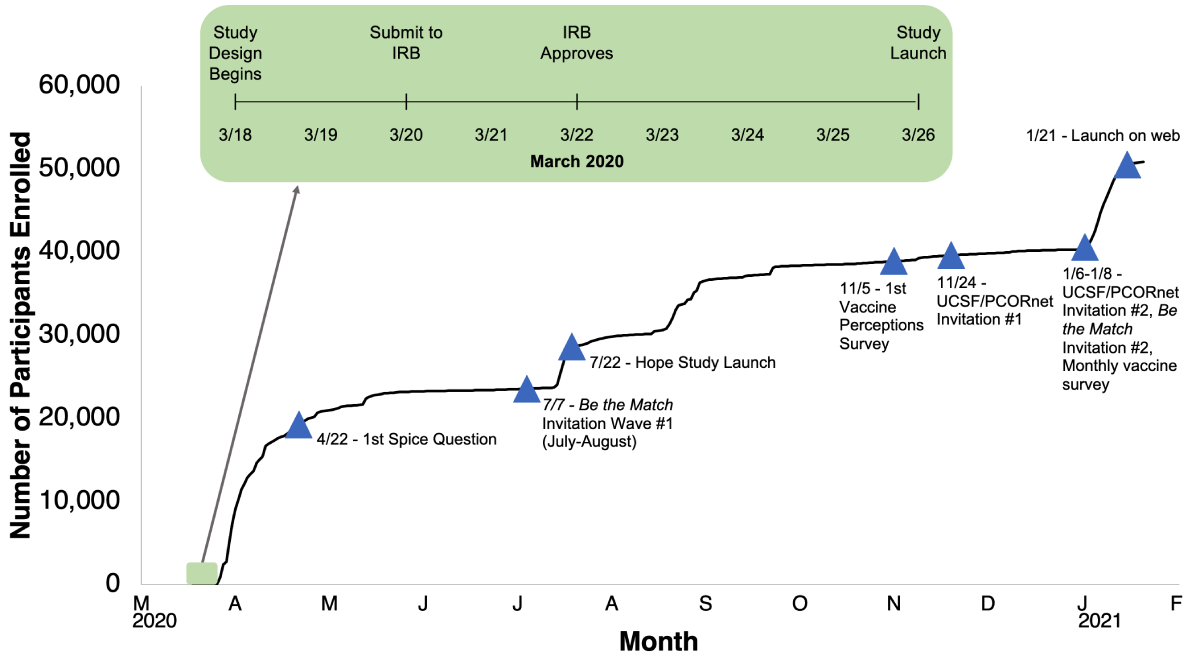
COVID-19 Citizen Science study development, enrollment, and iteration. IRB: institutional review board; PCORnet: National Patient-Centered Clinical Research Network; UCSF: University of California, San Francisco.

This report of an observational study is consistent with Strengthening the Reporting of Observational Studies in Epidemiology (STROBE) reporting guidelines. CCS is a cohort study on the Eureka Research Platform conducted through the Eureka mobile app (Figure 1) [18]. The CCS study was launched on March 26, 2020, after 8 days of intensive scientific and technical development utilizing established Eureka workflows (Figure 2). Participant enrollment and follow-up are ongoing and are expected to continue for years to investigate the long-term consequences of the COVID-19 pandemic. There are no prespecified enrollment targets or end dates for enrollment or completion. Participants must be 18 years of age or older, register for a Eureka account, have an iOS or Android smartphone, have a cell phone number, agree to participate in English, and be able to provide consent to participate in the study. After providing electronic consent to participate in the study, participants are asked to complete a baseline survey about demographics, medical conditions, medications, and behaviors through the study app. Participants can voluntarily provide permission to collect additional data from their smartphones, including geolocation and, among iOS users, HealthKit data (providing this data is optional and does not otherwise preclude study participation). Participants then complete daily, weekly, and monthly surveys through the study app. Surveys are written in lay language meeting Flesch-Kincaid criteria for an eighth-grade reading level [19]. The study does not currently include any interventions. On January 21, 2021, we also launched a web version of the study to enable participation options for people without a mobile phone or with concerns about downloading an app. The study is only available in English.

CCS is an entirely digital study that is hosted on the Eureka Research Platform, which has standard elements for electronic consent, surveys, and feedback that can be customized for individual studies. Studies on the Eureka Research Platform follow rapid development cycles that enable swift progression through study concept, user-centered design, programming, software quality assurance, and deployment to production. Additionally, study design and content are not static, but undergo iterative revisions in response to participant feedback, research collaborations, new scientific or public health findings, and newly developed research questions arising in the course of the research. Within the participant study app, questions are presented as one question per screen. The participant has the opportunity to go back and change responses within a survey, but they cannot change responses within the app after submitting the survey.

The Eureka platform and the CCS study were reviewed and approved by the UCSF Institutional Review Board (IRB) (#17-21879). Due to pre-existing Eureka IRB protocols and institutional prioritization of COVID-19 research, we received CCS study-specific IRB approval in under 3 days. All participants provide electronic consent to participate in the study. Some partner studies also include additional consent through Docusign. There were no monetary incentives for participation in the main study. Some partner studies include monetary incentives for participation to enhance participation in underrepresented groups.

### Recruitment

Study participants are recruited through multiple mechanisms, which not only allows broad study recruitment but also enables answering targeted research questions in specific populations through collaboration with research partners.

The Eureka app is publicly available on the Apple App Store and Google Play Store; anyone who downloads and installs the Eureka app and meets the CCS study enrollment criteria can participate. No payment is required, and no monetary incentives are provided. The Eureka app hosts many studies; some studies (such as CCS) are visible to anyone with the app and other studies are only visible with specific deep links. The CCS study is broadly advertised through press releases and articles in print and digital media, including social media. To date, advertisement has been conducted organically without paid or targeted ads, but rather by word of mouth, social media sharing, coverage by news outlets or other external content creators, and outreach to interested organizations. Digital advertisements include deep links that take participants directly to the app store to download and then open the Eureka app to specifically enroll in the CCS study. Participants may see opportunities to join other studies, but only after they finish their CCS-related study activities. Advertisements can also include “text backs” wherein a word (such as “COVID”) is texted to a 5-digit SMS short code (such as 41411), resulting in an immediate text that provides the deep link. A similar mechanism also supports QR-code mediated recruitment. Referral codes also support tracking the recruitment source, such as from a specific article or video, allowing optimization of recruitment methods. Lastly, Eureka users who have previously downloaded the app may discover the CCS study in the app without external prompting.

We are also able to recruit study participants from other Eureka platform studies. For example, we sent emails to Health eHeart participants to invite them to participate in the CCS study. Recruitment email versions were tested, with two versions of emails sent to 71,669 prospective participants (randomized 1:1) over 2 days in the first phase of recruitment. We then selected the more successful email version, defined by the number of consented participants who received each, to send to later phases of recruitment to increase the likelihood of participation.

CCS also employs a partner study model for recruitment. Through this model, an individual in a selected population is invited to participate in the CCS study using a unique participant code. This code enables the data that are collected through the CCS to be linked with the participant’s data from another program, registry, or research study, with data sharing under the auspices of a partner-specific consent that is presented during study onboarding prior to the general CCS consent. Each partner maintains its own IRB approval distinct from the core CCS study and Eureka platform approvals. One example of partner study recruitment is the invitation of *Be The Match* registry participants to join the CCS study. *Be The Match* registry participants, who have registered as potential bone marrow donors and undergone HLA antigen typing [20], were emailed a unique link to enroll in the CCS study. Researchers will then be able to study whether HLA antigen types are related to COVID-19 risk. A second example of partner study recruitment is through health systems willing to deliver invitations to their patients [21]. Health systems email participants an invitation to participate with a unique link. The participant first provides partner consent to link their standardized electronic health record data to their CCS data before proceeding to CCS enrollment and consent. Researchers will then be able to link patient-reported data from the CCS study with electronic health record data (eg, from health systems participating in the National Patient-Centered Clinical Research Network [PCORnet]) [22].

Recruitment can also occur with partner studies through two-way recruitment and data-sharing. The CCS study partnered with the HOPE COVID-19 (Health Outcome Predictive Evaluation for COVID-19) study investigating the impact of the COVID-19 pandemic on pregnancy [23]. CCS study participants who may be eligible are sent invitations to participate in the HOPE COVID-19 study, and participants in the HOPE COVID-19 study are sent invitations to participate in the CCS study. Each study can link data for mutual participants to generate richer data in a specific topic area and limit duplication of study questions. Through this IRB-approved process, participants can provide consent to data sharing across studies.

### Engagement

Enhancing engagement in digital clinical studies is a challenge, and we have employed several engagement approaches to date. Since the study includes daily surveys, we send a push notification to participants on a daily basis at the time the survey becomes available. In order to address various time zones as well as work schedules, the most convenient time of day for each participant is automatically inferred by sending subsequent notifications at 24-hour intervals following completion of the baseline set of surveys. For weekly and monthly surveys, we send both a push notification and a delayed SMS text message (which is not sent if the activities are completed before the delay) with a link to the Eureka app. The SMS text message is deliberately utilized less frequently than the mobile app-based notification because it is more intrusive, potentially resulting in heightened response rates and incurring greater participant burden. These surveys are sent at 7-day intervals from the completion of the baseline survey.

Additionally, because one motivation for this study is citizen participation in science, we actively promote feedback of study information to participants. We post study updates to a public website [24] that is linked within the study app and periodically send participants messages with study updates (approximately quarterly).

**Figure 3:**
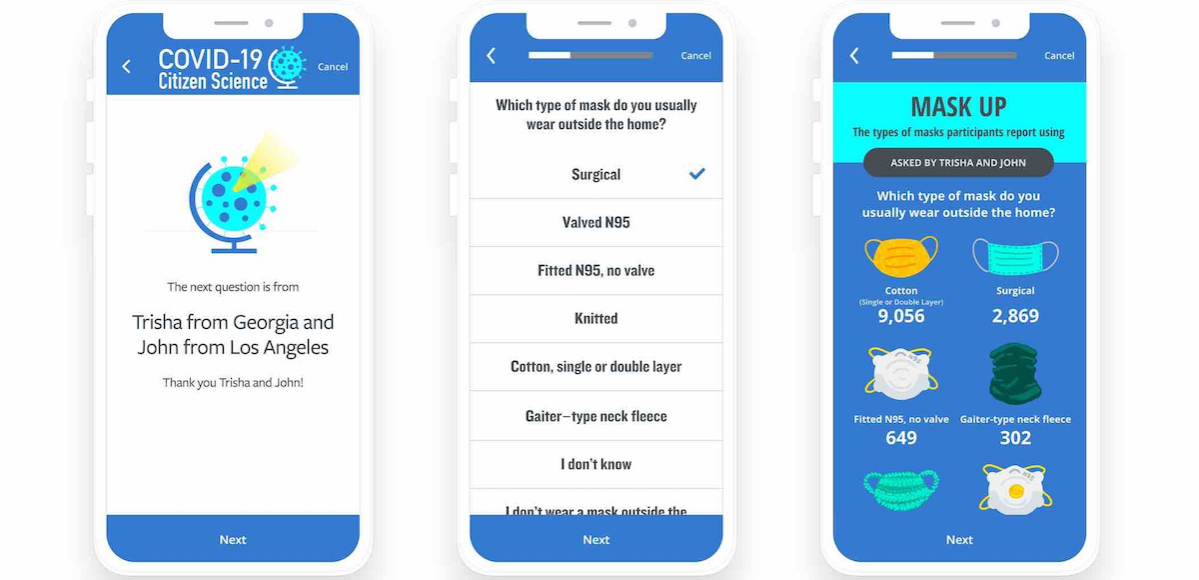
Example of participant-proposed “spice question” engagement method on the COVID-19 Citizen Science study app. Screenshots depict an introductory screen, a survey question, and a subsequent report of results delivered to the survey participants.

A novel engagement technique in this study is the *Spice Question* (Figure 3). Because the study includes repetitive daily surveys, we instituted a Spice Question that is added to the survey approximately once a week. The day-of-week that a Spice Question is sent is intentionally variable to avoid predictability to invoke intermittent reinforcement, potentially employing similar psychological engagement as a random slot machine [25]. This is also a mechanism to rapidly incorporate research questions posed to the entire cohort at one point in time that may be addressed with a single question. To promote the *citizen science* aspect of the study, we asked study participants to submit suggestions for Spice Questions through a survey mechanism. After review and edits as needed to avoid replication, limit questions to those that are answerable via survey, and to assure the question is delivered in easily understandable language, we then present the participant-proposed Spice Question to study participants. For these questions, we indicate that the question originated from a study participant, and we include the following details of the participant that contributed the question: first name and town or city of residence along with the state or country (if outside the US) name (Figure 3). Spice Questions are often the topic of website update posts to further emphasize citizen science engagement in research questions, participation, and sharing of results

Study participants are also able to request support or submit suggestions for study improvement through the app or email to the study staff. These messages are managed through Zendesk, which allows us to rapidly triage and respond to participant messages (median response time is 14.3 hours). Participant suggestions are routinely used to refine survey questions and identify technical bugs.

### Data Collection and Management

Study data can be collected from participant-reported responses within the study app, smartphone data, connected devices and services, and partner data such as electronic health record data. Additionally, these data can be supplemented by information from public sources, including data on regional health care policies, and public data on COVID-19 cases and deaths, such as data through the New York Times GitHub server. Study data are stored on private, secure, HIPAA (Health Insurance Portability and Accountability Act)–compliant cloud-based servers with access restricted only to authorized study personnel.

Surveys include baseline, daily, weekly, and monthly surveys (Table 1 and Multimedia Appendix 1). At baseline, participants are asked about their past COVID-19 test results, demographics, medical history, medications, and behaviors. Daily surveys ask about symptoms and interactions outside of the household. These questions were asked daily to enable the study team to capture rapidly changing symptoms. Weekly surveys ask about new COVID-19 test results and community behaviors to promote response while the test results and behaviors are fresh in the participant’s mind. Monthly surveys ask about depressive symptoms and anxiety symptoms in validated questionnaires that are designed to capture symptoms experienced by the participants over the past 2 weeks. Through the course of the study, additional surveys have been added, such as surveys about vaccine perceptions and receipt of a COVID-19 vaccine. Surveys were designed to ask participants about known COVID-19–related symptoms, risk mitigation behaviors, disease occurrence, and potential demographic characteristics and medical conditions that may confound associations between exposures and outcomes. When possible, the study uses validated questionnaires to enhance the validity and interpretation of results.

**Table 1:** COVID-19 Citizen Science study survey schedule.

Survey item	Baseline	Daily	Weekly	Monthly	One-time
Demographics	✓				
Medical history	✓				
Medications	✓		✓		
MacArthur Scale of Subjective Social Status [26]	✓				
Patient Health Questionnaire–8 (omits suicidal ideation) [27]				✓	
General Anxiety Disorder Questionnaire–7 [28]				✓	
COVID-19 symptoms, interactions outside of household	✓	✓			
Community behaviors eg, (large events, restaurants, bars, gyms)			✓		
COVID-19 testing and results	✓		✓		
Vaccine perceptions					✓
Vaccine receipt	✓			✓	
Spice questions					✓

Smartphone and device data include data collected through Apple HealthKit, as authorized by the participant to be shared with the CCS study team (Multimedia Appendix 2). We also collect geolocation data to allow us to identify and measure time spent at places of interest. Through a partnership with COVIDSEEKER [29], a novel tool being tested at UCSF, we invite CCS participants to contribute retrospective geolocation data to identify locations of possible COVID-19 exposures.

### Statistical Analysis

Due to the rapidly changing nature of the COVID-19 pandemic, we did not prespecify a sample size or statistical analyses for the study. Each research project that researchers wish to conduct using data from the CCS study will formulate a statistical analysis plan prior to analyzing data and register that analysis with the CCS study team. Examples include the following studies: (1) “Characteristics and behaviors associated with prevalent SARS-CoV-2 infection” [30], (2) “Factors associated with access to and timing of coronavirus testing among US adults after onset of febrile illness” [31], and (3) “Predictors of incident viral symptoms ascertained in the era of COVID-19” [32].

## Results

**Figure 4:**
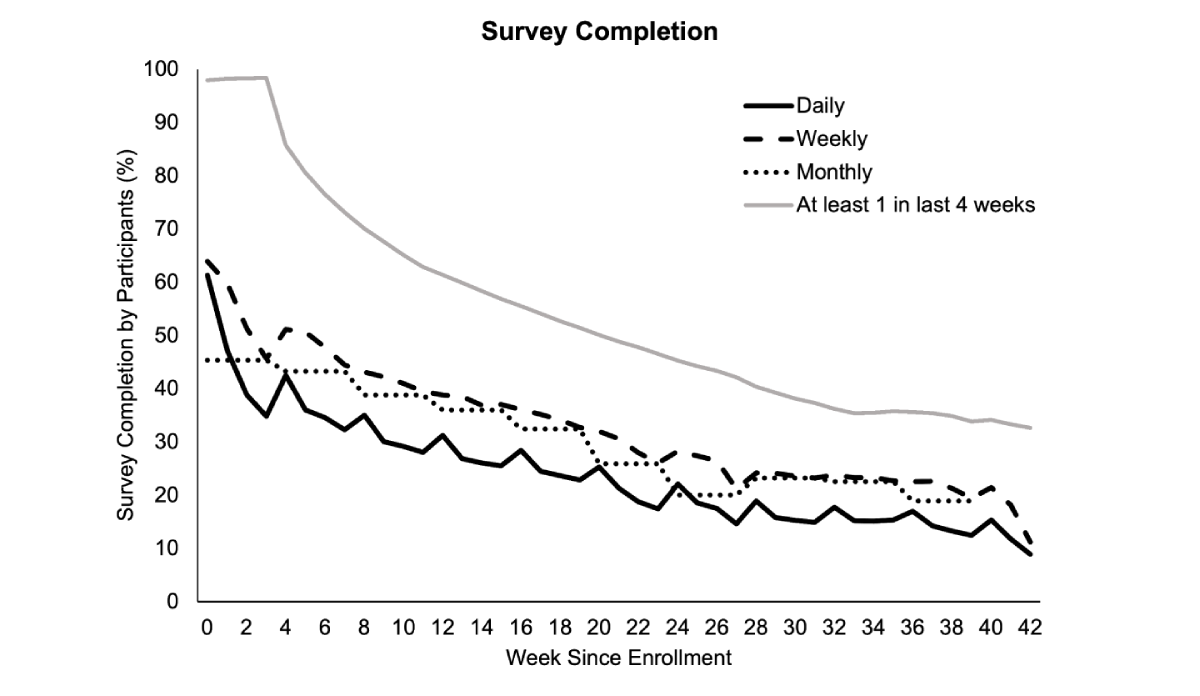
Survey completion metrics in the COVID-19 Citizen Science study.

**Figure 5:**
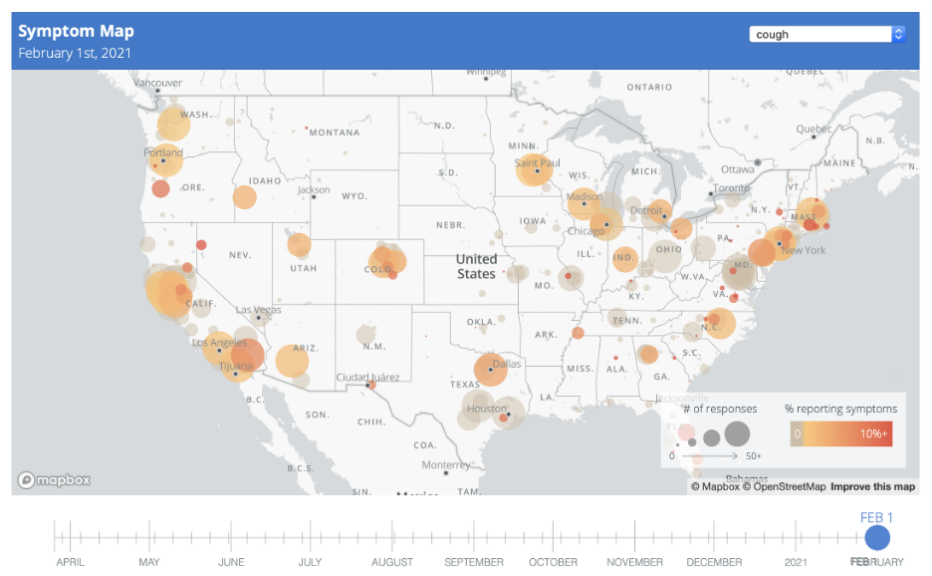
COVID-19 Citizen Science study symptom map (see Multimedia Appendix 4 for details).

Participants from every state in the USA and 99 countries have enrolled in the CCS study (Multimedia Appendix 3). Baseline characteristics of participants enrolled as of January 14, 2021, are presented in Table 2. Geolocation data were contributed by 78.3% (36,116/46,106) of participants, and HealthKit data were contributed by 34.8% (16,044/46,106) of all participants. Although the percentage of participants completing surveys decreases over time since enrollment (Figure 4), over the lifetime of the study, an average of 59% of participants have completed at least 1 survey in the past 4 weeks. The study publishes real-time results on a public website [24] that is linked from the participant study app. These results include updates on numbers of participants, interactive maps of worldwide participation, and patient-reported symptoms (Figure 5; Multimedia Appendix 4) [33], and blog posts with charts illustrating participant responses to study questions. Ongoing efforts include creating a dashboard to display historic and real-time results and future predictions about COVID-19 trends based on data contributed by CCS participants and publicly available data.

**Table 2:** Baseline characteristics of participants in the COVID-19 Citizen Science study as of January 14, 2021 (N=46,106).

Characteristic^a^		Value
**Age at consent (years), n (%) (n=46,106)**		
	18-39	20,897 (45.3)
	40-64	21,475 (46.6)
	≥65	3734 (8.1)
**Sex at birth, n (%) (n=42,902)**		
	Male	13,437 (31.3)
	Female	29,391 (68.5)
	Decline	74 (0.2)
**Gender identity, n (%) (n=42,890)**		
	Male	13,289 (31)
	Female	28,904 (67.4)
	Transgender woman	53 (0.1)
	Transgender man	92 (0.2)
	Genderqueer	327 (0.8)
	Other	137 (0.3)
	Decline	98 (0.2)
**Race, n (%) (n=42,418)**		
	Black or African American	875 (2.1)
	White	38,086 (89.8)
	Asian	3150 (7.4)
	Native Hawaiian or Pacific Islander	131 (0.3)
	American Indian or Alaska Native	569 (1.3)
	Other or don’t know	1516 (3.6)
Hispanic ethnicity, n (%) (n=42,902)		3305 (7.7)
Subjective social status, median (IQR) (n=42,898)		7 (6-8)
**Educational attainment, n (%) (n=42,885)**		
	High school or less	1951 (4.5)
	Some college	6864 (16)
	Bachelor’s	15,394 (35.9)
	Postgraduate	18,140 (42.3)
	Other, don’t know, or prefer not to state	536 (1.2)
Residence in USA, n (%) (n=43,905)		41,058 (93.5)
**Residence: USA region, n (%) (n=40,943)**		
	West	20,146 (49.2)
	Midwest	6662 (16.3)
	Northeast	5723 (14)
	South	8412 (20.5)
**Exercise, n (%) (n=43,819)**		
	Never	2775 (6.3)
	Less than once per month	4146 (9.4)
	Once per month to once per week	5857 (13.3)
	Once per week	5792 (13.2)
	1 to 4 times per week	13,275 (30.2)
	4 or more times per week	11,900 (27.1)
	Other	146 (0.3)
Alcohol consumption (drinks/week), median (IQR) (n=27,649)		2 (0-5)
Current smoking, n (%) (n=41,604)		2385 (5.7)
Current e-cigarette use, n (%) (n=41,682)		1408 (3.4)
**Medical condition, n (%)**		
	Hypertension (n=42,783)	7969 (18.6)
	Diabetes (n=42,780)	1676 (3.9)
	Coronary artery disease (n=42,781)	979 (2.3)
	Myocardial infarction (n=42,781)	402 (0.9)
	Congestive heart failure (n=42,781)	266 (0.6)
	Stroke or transient ischemic attack (n=42,783)	520 (1.2)
	Atrial fibrillation (n=42,780)	1128 (2.6)
	Sleep apnea (n=42,781)	4226 (9.8)
	Chronic obstructive pulmonary disease (n=42,782)	682 (1.6)
	Asthma (n=42,780)	4414 (10.3)
	Cancer (active) (n=42,779)	1272 (2.9)
	Immunodeficiency (n=42,783)	867 (2)
	HIV (n=42,783)	179 (0.4)
	Anemia (n=42,780)	4530 (10.6)
	Pregnant (n=42,781)	515 (1.2)
Tested positive for COVID-19 before baseline, n (%) (n=43,905)		1214 (2.8)

^a^Not all participants provided responses to all survey questions, so the denominator for each characteristic differs.

## Discussion

The CCS study was rapidly designed and launched, facilitated by the existing infrastructure, workflows, and approvals of the NIH-funded Eureka Research Platform. Consequently, the CCS study team was able to rapidly enroll and engage large numbers of participants to generate real-time research results.

Other digital cohort studies have been launched during the pandemic to track symptoms and disease occurrence. The COVID Symptom Study has enrolled millions of participants, primarily in the United Kingdom and the United States to track symptoms of COVID-19. The COVID Symptom Study cohort has a similar proportion of female participants and less racial and ethnic diversity, with 92.5% of the cohort identifying as White and only 0.5% identifying as Hispanic [3,34]. COVIDENTIFY is a digital cohort study that aims to determine whether smartphone and smartwatch data can predict COVID-19. The study reported similar demographic and gender representation in early study participants [35].

The strengths of our study include the incorporation of real-time feedback data to participants through our study website and engagement with participants through novel engagement methods, such as *Spice Question*s. Additionally, our partnerships with other studies will have implications for studying the association of HLA antigen types with COVID-19 and analysis of the combination of patient-reported and device data with electronic health record data. These studies may yield new knowledge about the biology of COVID-19 and improve our understanding of COVID-19 symptoms, risk mitigation behaviors, and disease occurrence within communities.

Studies relying on digital methods for recruitment and participation often raise concerns about diverse, inclusive, and equitable participation [5]. Indeed, this study has limitations with regard to nonrandom geographic distribution and low participation among traditionally underrepresented research populations. Although approaching design with user-centered design methods can reduce potential barriers, additional efforts are needed to promote digital clinical studies that are diverse, inclusive, and equitable. Ongoing work on the Eureka Research Platform will enable a Spanish-language version of the CCS study. Additionally, we anticipate generating insights from targeted recruitment at clinical sites with connected electronic health records that will seek to recruit participants identifying as racial or ethnic minorities, since these groups have been disproportionately affected by the COVID-19 pandemic. Recruitment methods will include additional outreach through letters, phone calls, and incentives for enrollment and participation [22]. Prior research on engagement of diverse communities has also demonstrated that individual outreach at community events may also facilitate participation in digital studies [12]. Beyond individual outreach, providing technology training may also be a strategy to enable participation among individuals with limited digital literacy or other barriers related to social determinants of health [8]. Future research is needed to better understand how to recruit, enroll, and engage participants in digital clinical studies to promote diverse, inclusive, and equitable health care and research.

The findings from this study will be subject to potential limitations. Although the study has recruited participants from 99 countries, because of the low levels of participation outside of the United States, the ability to conduct analyses based on country-specific characteristics such as development, income, and education levels may be limited. Because this is an observational study, we may not be able to make causal statements about associations between exposures and outcomes. We are collecting an array of participant-reported measures that may help to address potential confounding bias, but we will not be able to eliminate potential confounding from unmeasured factors. Measurement bias may be observed in participant-reported outcomes. When possible, the study uses validated questionnaires to limit bias. Bias may also occur from potentially differential loss to follow-up of participants. The CCS study has an average of 59% continued participation in surveys, which is similar to a digital follow-up on surveys that have been observed in other digital cohort studies, even in well-established cohorts such as the Framingham Heart Study [2]. Nevertheless, the CCS study team will continue to make efforts to engage participants through real-time updates and novel engagement strategies, such as participant-proposed *Spice Questions*.

This study illustrates important advances in digital clinical studies, including an entirely digital study participation, targeted recruitment strategies, electronic consent, analytics for recruitment and engagement, recurrent and time-updated assessments, integration with smartphone-based measurements, connection with partner studies, novel engagement strategies such as participant-proposed questions, and feedback in the form of real-time results to participants. Continued innovation in digital clinical study methods represents the future of clinical research.

## Appendix

Multimedia Appendix 1 COVID-19 Citizen Science study surveys.

Multimedia Appendix 2 HealthKit data types used in the COVID-19 Citizen Science study.

Multimedia Appendix 3 Countries represented by study participants.

Multimedia Appendix 4 Video of patient-reported symptoms in the COVID-19 Citizen Science study.

## Abbreviations

CCS: COVID-19 Citizen Science
HIPAA: Health Insurance Portability and Accountability Act
HOPE COVID-19: Health Outcome Predictive Evaluation for COVID-19
IRB: institutional review board
PCORI: Patient-Centered Outcomes Research Institute
PCORnet: National Patient-Centered Clinical Research Network
STROBE: Strengthening the Reporting of Observational Studies in Epidemiology
UCSF: University of California, San Francisco
