# Supplementary material for: The COVID-19 Citizen Science Study: Protocol for a Longitudinal Digital Health Cohort Study
Source: JMIR Res Protoc. 2021 Aug 30;10(8):e28169. doi: 10.2196/28169 (PMC8407439; doi:10.2196/28169)
Supplement: Multimedia Appendix 1 [file resprot_v10i8e28169_app1.pdf]

Sections

Baseline Data Collection

Baseline Survey

Baseline Data Collection

In what country is your primary residence?

What is the ZIP code (if in the U.S.) or postal code of your primary residence?

Have you had any of the following symptoms since February 1, 2020 for more than 3 days in a row? CHECK ALL THAT APPLY

- ☐ A scratchy throat
- ☐ A painful sore throat
- ☐ A cough (worse than usual if you have a baseline cough)
- ☐ A runny nose
- ☐ Symptoms of fever or chills
- ☐ A temperature greater than 100.4 °F or 38.0 °C
- ☐ Muscle aches (worse than usual if you have baseline muscle aches)
- ☐ Nausea, vomiting or diarrhea
- ☐ Shortness of breath
- ☐ Unable to taste or smell
- ☐ Red or painful eyes
- ☐ None of the above

Have you ever been tested for the novel coronavirus, the virus that causes COVID-19 (either a test to detect the virus for active infection or the antibody to detect past infection)?

- ○ Yes
- ○ No

Other

Was it a test for active infection (virus) or past infection (antibody to the virus)? (The test for active infection usually uses a swab or saliva; the test for past infection usually uses blood.)

- Test for active infection (virus)
- Test for past infection (antibody to the virus)
- I had both kind of tests
- I don’t know

Do you think you previously experienced symptomatic infection due to COVID-19?

- Yes
- No

When did your symptoms start?

What symptoms did you have? CHECK ALL THAT APPLY

- [ ] A scratchy throat
- [ ] A painful sore throat
- [ ] A cough (worse than usual if you have a baseline cough)
- [ ] A runny nose
- [ ] Symptoms of fever or chills

- [ ] A temperature greater than 100.4 °F or 38.0 °C
- [ ] Muscle aches (worse than usual if you have baseline muscle aches)
- [ ] Nausea, vomiting or diarrhea
- [ ] Shortness of breath
- [ ] Unable to taste or smell
- [ ] Red or painful eyes
- [ ] Other

If other, please explain.

During the illness that you believe was due to COVID-19, were you tested for the flu?

- ( ) Yes
- ( ) No

What was the result?

- ( ) Positive for the flu
- ( ) Negative for the flu
- ( ) Other

Prior to the illness you believe was due to COVID-19, were you in physical

contact with someone else that tested positive for the disease?

- Yes
- No
- Other

Prior to the illness you believe was due to COVID-19, were you in physical contact with someone else with symptoms suggestive of COVID-19?

- Yes
- No
- Other

Prior to the illness you believe was due to COVID-19, had you traveled to a region known to have a high prevalence of COVID-19?

- Yes
- No
- Other

During the illness you believe was due to COVID-19, did you seek to receive a test for active COVID-19 infection?

- Yes
- No
- Other

What happened when you sought the coronavirus test?

- I did receive a test, and it was positive.
- I did receive a COVID-19 test for active infection, and it was negative.
- I did receive a COVID-19 test for
- I was evaluated by a healthcare

active infection, but do not know the results.

provider, but they did not believe the test was indicated.

I was evaluated by a healthcare provider and they wanted to order a test, but it was not available.

Other

Do you continue to have symptoms due to the illness you believe to be due to COVID-19?

Yes

No

On what date did you last experience symptoms?

Are there other reasons not covered by this survey that lead you to believe you have been infected with the novel coronavirus?

Yes

No

Other

Please explain.

About how many weeks ago was your test for active COVID-19 infection (virus)? Put 0 if this week.

weeks ago

About how many weeks ago was your test for past infection (antibody to the COVID-19 virus)? Put 0 if this week.

weeks ago

Do you know the result of your test for active COVID-19 infection (virus)?

- Yes, I was positive (the novel coronavirus WAS detected)
- Yes, I was negative (the novel coronavirus was NOT detected)
- Yes, the test was inconclusive
- No, not yet

Do you know the result of your test for past infection (antibody to the COVID-19 virus)?

- Yes, I was positive (antibody to COVID-19 WAS detected suggesting past exposure)
- Yes, I was negative (antibody to COVID-19 was NOT detected suggesting NO past exposure)
- Yes, the test was inconclusive
- No, not yet

Why was the test for active COVID-19 infection (virus) performed? CHECK ALL THAT APPLY

- [ ] I had symptoms concerning for COVID-19 infection (including hospitalization for COVID-19)
- [ ] I was exposed to someone with suspected or confirmed COVID-19
- [ ] Prior to a medical procedure or hospitalization that was unrelated to COVID-19
- [ ] It was offered through my healthcare provider as part of routine screening (not related to symptoms or pregnancy)

- [ ] It was part of screening for my pregnancy
- [ ] I am a healthcare worker and it is offered or mandated by my employer
- [ ] As part of a research study
- [ ] It was required by my work
- [ ] Part of a public health effort
- [ ] I obtained it on my own
- [ ] Not sure or other

Why was the test for past infection (antibody to the COVID-19 virus) performed? CHECK ALL THAT APPLY

- [ ] I had symptoms concerning for COVID-19 infection (including hospitalization for COVID-19)
- [ ] I was exposed to someone with suspected or confirmed COVID-19
- [ ] Prior to a medical procedure or hospitalization that was unrelated to COVID-19
- [ ] It was offered through my healthcare provider as part of routine screening (not related to symptoms or pregnancy)
- [ ] It was part of screening for my pregnancy
- [ ] I am a healthcare worker and it is offered or mandated by my employer

- [ ] As part of a research study
- [ ] It was required by my work
- [ ] Part of a public health effort
- [ ] I obtained it on my own
- [ ] Not sure or other

Which of the following describes your primary area of employment?

- ○ Healthcare
- ○ Education
- ○ Retail
- ○ Transportation
- ○ Arts, entertainment, and recreation
- ○ Hospitality and food services
- ○ Finance and insurance
- ○ Scientific and technical services
- ○ Utilities
- ○ Construction
- ○ Manufacturing
- ○ Other

Are you aware of any novel coronavirus (the virus causing COVID-19) infected individuals in your COUNTY (or local area equivalent if your area does not have counties)?

- Yes
- No

How worried are you that the health of you or your loved ones will be affected by the novel coronavirus (the virus causing COVID-19)?

- Extremely worried
- Very worried
- Somewhat worried
- A little worried
- Not worried at all

Has your local government issued or continued any of the following restrictions? CHECK ALL THAT APPLY

- School closures
- Restricted gatherings at (or closed) bars, restaurants, and/or theaters
- Restricted gatherings of a certain number of individuals
- Recommended working from home or not working
- Shelter in place (required to stay home except for essential activities)
- Other restrictions

How have your hand hygiene practices (washing hands and/or using hand sanitizer) changed since learning about the novel coronavirus (the virus causing COVID-19)?

- I wash or sanitize my hands MUCH MORE frequently than before
- I wash or sanitize my hands SOMEWHAT MORE frequently than before

- I wash or sanitize my hands A LITTLE MORE frequently than before
- I have not made any changes
- I wash or sanitize my hands A LITTLE LESS frequently than before
- I wash or sanitize my hands SOMEWHAT LESS frequently than before
- I wash or sanitize my hands MUCH LESS frequently than before

Have you sanitized your mobile phone (such as by using sanitizing wipes or hand sanitizer) since learning of the novel coronavirus (the virus causing COVID-19)?

- Yes
- No
- Other

For the next question, "household" is defined as the number of individuals who live with you. This would include yourself, as well as any other adults and children. Make sure to include family members as well as people who are not related to you. This would also include people who normally live in your household, but who are away traveling temporarily. If you’re on the mobile app, tap next to continue.

What is the number of individuals who, during the past month, normally live in your household. This would include yourself, as well as any other adults and children. Make sure to include family members as well as people who are not related to you. This would also include people who normally live in your

household, but who are away travelling temporarily.

How many separate rooms are in the place where you live? INCLUDE bedrooms, kitchens, living rooms, etc. EXCLUDE bathrooms, porches, balconies, foyers, halls, or unfinished basements.

Do any school-aged (K-12 or equivalent) children live with you?

- Yes
- No
- Other

Do you have a college-aged child (under the age of 25) who usually does not live in your home but who has returned home and is living in your house because of the coronavirus pandemic?

- Yes
- No

What date did they return? (Your best guess is fine.)

MM/DD/YYYY

What school were they attending?

School

Where is the school located?

Do you live with or have continued regular in-person contact with an elderly person (over 65 years of age) or someone susceptible to illness (being immunocompromised or having a pre-existing medical condition)?

- ○ Yes
- ○ No
- ○ Other

Do you have any pets at home?

- ○ Yes
- ○ No
- ○ Other

What pets live with you (CHECK ALL THAT APPLY):

- ☐ Dog(s)
- ☐ Cat(s)
- ☐ Bird(s)
- ☐ Reptile(s)
- ☐ Other

Did you have a flu shot (influenza vaccine) in the past year?

- ○ Yes
- ○ No
- ○ Other

Have you had cold or flu symptoms (enough that you would say that you had a cold or the flu) in the past year?

- ○ Yes
- ○ No

How many cold or flu illnesses in the past year were associated with a fever

(Temperature > 101.3 F or > 38.5 C)?

- None
- 1-3
- 4-6
- More than 6

When was the last one?

[ ] weeks ago

How many cold or flu illnesses in the past year were NOT associated with a fever (Temperature > 101.3 F or > 38.5 C)?

- None
- 1-3
- 4-6
- More than 6

When was the last one?

[ ] weeks ago

On average, how often have you exercised (enough to breathe heavily and/or sweat) over the past year?

- Never or rarely
- Less than once a month
- More than once a month but less than once a week
- About once a week
- More than once a week but less than 4 times a week
- 4 or more times a week
- Other

IN THE PAST WEEK: How many drinks of alcohol (one drink = one standard glass of wine, can of beer, or shot of hard liquor) did you consume?

drinks

Click here to finish

Sections

Baseline Data Collection

Demographics Survey

Baseline Data Collection

What sex were you assigned at birth?

- Male
- Female
- Prefer not to disclose

How would you describe your current gender identity?

- Male
- Female
- Transgender Woman (Male-to-Female)
- Transgender Man (Female-to-Male)
- Genderqueer
- Another Gender Identity
- Decline to state

What gender identity do you identify with? (Optional)

What is your racial background? CHECK ALL THAT APPLY.

- Black or African American
- White
- Asian (including South Asian and Asian Indian)
- Native Hawaiian or Pacific Islander
- American Indian or Alaska Native
- Some other race
- Don't know

What is your Asian background?

- Chinese
- Filipino
- Asian Indian
- Japanese
- Korean
- Vietnamese
- Other Asian or Mix

What is your Pacific Island background?

- Native Hawaiian
- Samoan
- Guamanian or Chamorro
- Other Pacific Islander or Mix

This is a question about ethnicity, rather than race, as used in the US Census. For example, someone may be of white race and Hispanic ethnicity or black race and Hispanic ethnicity. Tap next to continue.

Are you of Hispanic, Latino or Spanish origin or ancestry?

- No
- Yes: Mexican, Mexican American or Chicano
- Yes: Puerto Rican
- Yes: Cuban
- Yes: Other or Mixed Hispanic, Latino or Spanish origin
- Don't know
- Prefer not to state

Think of this ladder as representing where people stand in your country. At the top of the ladder are the people who are the best off -- those who have the most money, the most education and the most respectful jobs. At the bottom are the people who are the worst off -- who have the least money, least education, and least respectful jobs or no job. The higher up you are on the ladder, the closer you are to the people at the very top; the lower you are, the closer you are to the people at the very bottom. Tap next to continue.

Where would you place yourself on this ladder?

What is the highest level of education you have achieved?

- No formal schooling
- Some school, but did not graduate high school
- High school diploma or equivalency (e.g., GED)
- Associate degree (e.g., junior college)
- Some college, but did not graduate college
- Bachelor's degree
- Master's degree
- Doctorate (PhD)
- Professional doctorate (MD, JD, DDS, etc.)
- Other
- Don't know
- Prefer not to state

Click here to finish

Sections

Monthly Surveys

Anxiety Survey

Monthly Surveys

Becoming easily annoyed or irritable.

- Not at all
- Several days
- More than half the days
- Nearly every day

Over the last two weeks, how often have you been bothered by the following problems? Tap next to continue.

Feeling nervous, anxious, or on edge.

- Not at all
- Several days
- More than half the days
- Nearly every day

Not being able to stop or control worrying.

- Not at all
- Several days
- More than half the days
- Nearly every day

Worrying too much about different things.

- Not at all
- Several days
- More than half the days
- Nearly every day

Trouble relaxing.

- Not at all
- Several days

More than half the days | Nearly every day

Being so restless that it is hard to sit still.

Not at all | Several days

More than half the days | Nearly every day

Feeling afraid as if something awful might happen.

Not at all | Several days

More than half the days | Nearly every day

Click here to finish

Sections

Baseline Data Collection

Your Medical Conditions

Baseline Data Collection

Have you ever been told by a doctor or nurse that you have, or have been treated for, any of the following conditions (in the past or currently)? Tap next to continue.

High blood pressure or hypertension (except that occurred during pregnancy and did not last after pregnancy)?

- Yes
- No
- Don't know

Diabetes? Do not include pre-diabetes.

- Yes
- No
- Don't know

Coronary artery disease (blockages in your heart vessels) or angina (chest pain)?

- Yes
- No
- Don't know

A heart attack (myocardial infarction)?

- Yes
- No
- Don't know

Congestive Heart failure (CHF, Heart Failure)?

- Yes
- No
- Don't know

Stroke or TIA (Transient Ischemic Attack or Mini-Stroke)?

- Yes
- No
- Don't know

Atrial fibrillation (Afib, AF)?

- Yes
- No
- Don't know

Sleep apnea (obstructive sleep apnea, OSA)?

- Yes
- No
- Don't know

COPD (emphysema, chronic bronchitis, obstructive pulmonary disease)?

- Yes
- No
- Don't know

Asthma, to the point that you use inhalers daily or have been to the hospital for your asthma?

- Yes
- No

Don't know

Cancer (including leukemia or lymphoma) undergoing active treatment?

Yes

No

Don't know

Immunodeficiency (NOT including HIV)?

Yes

No

Don't know

Chronic HIV infection?

Yes

No

Don't know

Anemia or other blood disorder (do not include leukemia or lymphoma)?

Yes

No

Don't know

Are you currently pregnant?

Yes

No

Don't know

Click here to finish

Your Smoking History

Sections

Baseline Data Collection

Baseline Data Collection

Have you ever smoked a cigarette, even one or two puffs?

- Yes
- No
- Don't know
- Refuse to answer

Have you smoked cigarettes in the past 30 days?

- Yes
- No
- Refuse to answer

About how many days have you smoked a cigarette in the past 30 days?

On average, how many cigarettes per day have you smoked in the past 30 days (use 1 if less than one)

cigarettes per day

Have you ever smoked a cigar, cigarillo, or tobacco product other than cigarette, even one or two puffs?

- Yes
- No
- Don't know
- Refuse to answer

Have you smoked a cigar, cigarillo, or tobacco product other than a cigarette in the past 30 days?

Yes | No

Don't know | Refuse to answer

About how many days have you smoked a cigar, cigarillo, or tobacco product other than cigarette in the past 30 days?

[ ] days

On average, how many cigar, cigarillo, or tobacco product (other than cigarettes) per day have you smoked in the past 30 days (use 1 if less than one)?

[ ]

Have you ever used an electronic nicotine product (e-cigarette, vape nicotine), even one or two puffs?

Yes | No

Don't know | Refuse to answer

Have you used an electronic nicotine product in the past 30 days?

Yes | No

Don't know | Refuse to answer

About how many days did you use it in the past 30 days?

[ ] days

How many puffs from an e-cigarette do you typically take over the past 30

days?

How much did you spend on electronic delivery products in the past 30 days?

Dollars

Have you smoked or vaped marijuana, even one or two puffs?

- Yes
- No
- Don't know
- Refuse to answer

Have you smoked or vaped marijuana in the past 30 days?

- Yes
- No
- Don't know
- Refuse to answer

How many days did you smoke or vape marijuana in the past 30 days?

Days

Click here to finish

Baseline Vaccine Survey

Sections

baseline_vaccine_survey

baseline_vaccine_survey

Have you ever received a COVID-19 (SARS-CoV-2) vaccine?

- Yes
- No
- I don't know

Where did you get your COVID-19 vaccine?

- In a research study or clinical trial
- A doctor's office, clinic or hospital (not part of a research study or clinical trial)
- A pharmacy (Walgreens, CVS, other standalone pharmacy)
- A grocery store, supermarket or other store (Walmart, Target, etc)
- A health fair or other public event
- Public health department
- At my home (someone came to administer it to me)
- At my workplace
- At a school
- Somewhere else

Where did you get your COVID-19 vaccine?

How many vaccine doses have you received?

- 1 dose
- 2 doses

Other

I don't know

What was the date of your first COVID-19 vaccine (OK to guess if unsure)?

What was the date of your second COVID-19 vaccine (OK to guess if unsure)?

What brand of COVID-19 vaccine did you receive?

I don't know

Pfizer/BioNTech

Moderna

AstraZeneca/Oxford University

Sinovac

Johnson & Johnson

Novavax

Inovio Pharmaceuticals

Sanofi/GlaxoSmithKline

Other

Please enter the brand of your COVID-19 vaccine:

Have you experienced any of the following potential side effects after receiving your COVID-19 vaccine? Select all that apply.

Fever

Chills

- ☐ Fatigue
- ☐ Sore/scratchy throat
- ☐ Muscle pain
- ☐ Joint pain
- ☐ Headache
- ☐ Other pain
- ☐ Redness/swelling at the injection site
- ☐ Rash other than at the injection site
- ☐ Allergic reaction/anaphylaxis
- ☐ Other
- ☐ None of the above

Please describe the side effect(s).

[ ]

When did your side effect(s) start?

[ ] days after getting the vaccine

How many days did your side effect(s) last?

[ ] days

Rate the severity of your side effect(s)

- ○ Very Mild
- ○ Mild
- ○ Moderate
- ○ Severe
- ○

Very Severe

Do you plan to get a COVID-19 vaccine?

- Yes, definitely
- Yes, very likely
- Not sure
- No, probably not
- No, definitely not

What makes you MORE likely to receive a COVID-19 vaccine? Select all that apply.

- Concern for your own health
- Concern for health of your family or others
- Desire to return to pre-COVID way of life (e.g. work, school, economy)
- Confidence that it will work (effectiveness)
- Convenience/easily available
- Workplace requirements
- Religious reasons
- Political reasons
- Trust in your healthcare provider
- Information found in the news (TV, newspaper, radio, internet)
- Information found on social media (Facebook, Twitter)

- [ ] Other
- [ ] None of the above

What makes you LESS likely to receive a COVID-19 vaccine? Select all that apply.

- [ ] My risk of getting COVID-19 is low
- [ ] I think I've already had COVID-19
- [ ] If I did get COVID-19 I wouldn't suffer bad consequences
- [ ] My other medical condition(s)
- [ ] Concern that it won't work
- [ ] Concerns about side effects from the vaccine
- [ ] Too busy
- [ ] Concerns about difficulty paying for it

- [ ] Concerns about difficulty finding a place to get it
- [ ] Dislike of needles/shots
- [ ] Religious reasons
- [ ] Political reasons
- [ ] Information found in the news (TV, radio, newspaper, internet)
- [ ] Information found in social media (Facebook, Twitter)
- [ ] Other
- [ ] None of the above

Of the groups or persons below, whose recommendation on whether or not to receive a COVID-19 vaccine matters to you? Select all that apply.

- [ ] Family and friends
- [ ] My personal doctor
- [ ] Scientific community (doctors, researchers)
- [ ] Pharmaceutical industry (vaccine manufacturers)
- [ ] Governmental health
- [ ] The President of the United

organizations (e.g. FDA, CDC)

States

☐ High-profile celebrities or actors

☐ Local community or religious leaders

☐ Other

Please enter whose recommendation on COVID-19 vaccination matters to you?

Do you know where you would go to receive a COVID-19 vaccine?

○ Yes

○ No

If you were going to get a COVID-19 vaccine, where would you feel comfortable getting it? Select all that apply.

☐ In a research study or clinical trial

☐ A doctor’s office, clinic or hospital (not part of a research study or clinical trial)

☐ A pharmacy (Walgreens, CVS, other standalone pharmacy)

☐ A grocery store, supermarket or other store (Walmart, Target, etc)

- [ ] A health fair or other public event
- [ ] Public health department
- [ ] At my home, if someone came to administer it to me
- [ ] At my workplace
- [ ] At a school
- [ ] Nowhere - I don’t plan to get a vaccine
- [ ] Somewhere else

Where else would you feel comfortable getting a COVID-19 vaccine?

Click here to finish

Sections

Daily Surveys

Daily COVID-19 Citizen Science Survey

Daily Surveys

IN THE PAST 24 HOURS: have YOU had any of the following (CHECK ALL THAT APPLY):

- ☐ A scratchy throat
- ☐ A painful sore throat
- ☐ A cough (worse than usual if you have a baseline cough)
- ☐ A runny nose
- ☐ Symptoms of fever or chills
- ☐ A temperature greater than 100.4 °F or 38.0 °C
- ☐ Muscle aches (worse than usual if you have baseline muscle aches)
- ☐ Nausea, vomiting or diarrhea
- ☐ Shortness of breath
- ☐ Unable to taste or smell
- ☐ Red or painful eyes
- ☐ None of the above

Did you seek medical care for these symptoms?

- ○ Yes
- ○ No

For the next two questions, "household" is defined as the number of individuals who live with you. This would include yourself, as well as any other adults and children. Make sure to include family members as well as people who are not related to you. This would also include people who normally live in your household, but who are away traveling temporarily. If you’re on the

mobile app, tap next to continue.

IN THE PAST 24 HOURS, has ANYONE (other than you) in your household had ANY of those symptoms? (scratchy/sore throat, cough, runny nose, fevers/chills/high temperature, muscle aches, nausea/vomiting/diarrhea, shortness of breath, unable to taste or smell, red or painful eyes)

- Yes
- No
- Not sure

IN THE PAST 24 HOURS, approximately how many people outside of your household did you interact with while they were within 6 feet? ("Interact" is loosely defined as talking, touching, or just being within 6 ft of someone for longer than 1 or 2 minutes).

________ people

Approximately what percent of those people were wearing masks, or were behind a shield?

________ %

Click here to finish

Weekly COVID-19 Citizen Science Survey

Sections

Weekly Surveys

Weekly Surveys

In the past week, have you received results of any tests that you had done for the novel coronavirus, the virus that causes COVID-19 (either a test to detect the virus for active infection or the antibody to detect past infection)?

- Yes
- No
- I got a test, but don't know the results

Do you know how you might get a coronavirus test if you needed one?

- Yes
- No

Was it a test for active infection (virus) or past infection (antibody to the virus)? (The test for active infection usually uses a swab or saliva; the test for past infection usually uses blood.)

- Test for active infection (virus)
- Test for past infection (antibody to the virus)
- I had both kind of tests
- I don't know

WHEN DID YOU TAKE THE TEST for active COVID-19 infection (virus) for which you received the results this week? (It's okay to guess if you are unsure.)

Do you know the result of your test for active COVID-19 infection (virus)?

- Yes, I was positive (the novel coronavirus WAS detected)
- Yes, I was negative (the novel coronavirus was NOT detected)
- Yes, the test was inconclusive
- No, not yet

WHEN DID YOU GET THE RESULTS from your test for active COVID-19 infection (virus)? (It's okay to guess if you are unsure).

Do you know how to get an appointment to talk with a doctor or healthcare provider?

- Yes
- No
- I don't know

How worried were you about losing your housing, income or employment because of your positive test result?

- Extremely worried
- Very worried
- Somewhat worried
- A little worried
- Not worried at all

Were you told to isolate yourself for a certain amount of time so you wouldn’t infect anyone else?

- Yes
- No
- I don't know

Were you provided access to resources (food, housing, compensation for lost income) so that you could isolate yourself?

- Yes
- No
- I don't know

Were you contacted by anyone to talk about “contact tracing” (finding other people who might have been exposed to you while you were sick)?

- Yes
- No
- I don't know

WHEN DID YOU TAKE THE TEST for past infection (antibody to the COVID-19 virus)? (It's okay to guess if you are unsure)

Do you know the result of your test for past infection (antibody to the COVID-19 virus)?

- Yes, I was positive (antibody to COVID-19 WAS detected suggesting past exposure)
- Yes, I was negative (antibody to COVID-19 was NOT detected suggesting NO past exposure)
- Yes, the test was inconclusive
- No, not yet

WHEN DID YOU GET THE RESULTS from your test for past infection (antibody to the COVID-19 virus)? (It's okay to guess if you are unsure)

Why was the test for active COVID-19 infection (virus) performed? CHECK ALL THAT APPLY

- [ ] I had symptoms concerning for COVID-19 infection (including hospitalization for COVID-19)
- [ ] I was exposed to someone with suspected or confirmed COVID-19
- [ ] Prior to a medical procedure or hospitalization that was unrelated to COVID-19
- [ ] It was offered through my healthcare provider as part of routine screening (not related to symptoms or pregnancy)
- [ ] It was part of screening for my pregnancy
- [ ] I am a healthcare worker and it is offered or mandated by my employer
- [ ] As part of a research study
- [ ] It was required by my work
- [ ] Part of a public health effort
- [ ] I obtained it on my own
- [ ] Not sure or other

Why was the test for past infection (antibody to the COVID-19 virus) performed? CHECK ALL THAT APPLY

- [ ] I had symptoms concerning for COVID-19 infection (including hospitalization for COVID-19)
- [ ] I was exposed to someone with suspected or confirmed COVID-19
- [ ] Prior to a medical procedure or hospitalization that was unrelated to COVID-19
- [ ] It was offered through my healthcare provider as part of routine screening (not related to symptoms or pregnancy)
- [ ] It was part of screening for my pregnancy
- [ ] I am a healthcare worker and it is offered or mandated by my employer

- [ ] As part of a research study
- [ ] It was required by my work
- [ ] Part of a public health effort
- [ ] I obtained it on my own
- [ ] Not sure or other

Over the past WEEK, how worried have you been that the health of you or your loved ones will be affected by the novel coronavirus (the virus causing COVID-19)?

- ○ Extremely worried
- ○ Very worried
- ○ Somewhat worried
- ○ A little worried
- ○ Not worried at all

Over the past WEEK, on average, how often have you washed or sanitized your hands?

- ○ More than 10 times per day
- ○ 5-10 times per day

2-4 times per day

About once per day

Less than once per day

Over the past WEEK, how many times have you visited a gym?

Over the past WEEK, how many times have you visited a restaurant (not for takeout)?

Over the past WEEK, how many times did you eat INSIDE a restaurant (not outdoor seating)?

Over the past WEEK, how many times have you visited a bar?

Over the past WEEK, how many times have you visited a movie theater?

Over the past WEEK, how many times have you visited a grocery store or pharmacy?

Over the past WEEK, how many times have you visited an event with more than 10 people?

Over the past WEEK, how many times have you exercised for more than 20

minutes (enough to breathe heavily and/or sweat)?

[ ]

Over the past WEEK, has your local government issued or continued any of the following restrictions? (CHECK ALL THAT APPLY)

- [ ] School closures
- [ ] Restricted gatherings at (or closed) bars, restaurants, and/or theaters
- [ ] Restricted gatherings of a certain number of individuals
- [ ] Recommended working from home or not working
- [ ] Shelter in place (required to stay home except essential activities)
- [ ] Wearing masks when out in public
- [ ] Other restrictions
- [ ] None of the above
- [ ] I don't know

Over the past WEEK, on average, how many hours did you sleep per night?

[ ] hours per night

Over the past week, how often did you wear a mask (any kind of covering over your mouth and nose) when you're out in public?

- ( ) Never
- ( ) Sometimes
- ( ) Most of the time
- ( ) Always
- ( ) I did not go out in public this past week

Click here to finish

Sections

Monthly Surveys

Monthly COVID-19 Citizen Science Survey

Monthly Surveys

Please answer the following for the period of the past 30 days. Tap next to continue.

What best describes your current main daily activities and/or responsibilities over the past 30 days?

- Working full time
- Working part-time
- Unemployed, laid off, or looking for work
- In school (full- or part-time student)
- Stay-at-home parent or keeping household
- Retired
- Disabled
- Prefer not to state

How much of your working time is currently performed at home?

- 100% of the time
- 75-99% of the time
- 50-74% of the time
- 25-49% of the time
- 1-24% of the time
- None

Has your income changed in the past 30 days?

- Yes, it has increased
- Yes it has declined

No, it is about the same

Prefer not to state

In the past 30 days, by what percentage has your income increased?

[ ] %

In the past 30 days, by what percentage has your income declined?

[ ] %

In the past 30 days, have you been unemployed?

Yes

No

Prefer not to state

For the next question, "household" is defined as the number of individuals who live with you. This would include yourself, as well as any other adults and children. Make sure to include family members as well as people who are not related to you. This would also include people who normally live in your household, but who are away traveling temporarily. If you’re on the mobile app, tap next to continue.

What is the number of individuals who, during the past month, normally live in your household?

[ ] people

How many separate rooms are in the place where you live? INCLUDE bedrooms, kitchens, living rooms, etc. EXCLUDE bathrooms, porches, balconies, foyers, halls, or unfinished basements.

[ ] rooms

How hard is it for you (and your family) to pay for the very basics like food, rent or mortgage, heating, etc over the past 30 days?

- Very hard
- Hard
- Somewhat hard
- Not very hard
- Don’t know
- Prefer not to state

Did you have difficulty making ends meet over the past 30 days?

- Frequently
- Occasionally
- Hardly ever
- Never
- Don’t know
- Prefer not to state

IN THE PAST WEEK: How many drinks of alcohol (one drink = one standard glass of wine, can of beer, or shot of hard liquor) did you consume?

________ drinks

Click here to finish

Sections

Monthly Surveys

Hospitalization Survey

Monthly Surveys

Have you been hospitalized (had an overnight stay in a hospital) in the past month or since the last time you answered?

- Yes
- No

How many days did you spend in the hospital over the past 30 days?

days

Have you been to the emergency room or Urgent Care (when you were NOT admitted to the hospital overnight) in the past 30 days or since the last time you answered?

- Yes
- No

How many times did you go to the emergency room or Urgent Care (when you were NOT admitted to the hospital overnight) in the past 30 days or since the last time you answered?

When were you discharged from the hospital (if more than one time, use most recent)?

MM/DD/YYYY

What was the main reason for your most recent hospitalization (you can look at the papers you received at discharge from the hospital)?

- Suspected COVID-19 infection
- Asthma
- Chronic obstructive pulmonary
- Pneumonia

disease

Common flu

Heart attack

Arrhythmias

Other

Please specify the main reason for your hospitalization.

When did you most recently visit the emergency department or Urgent Care?

MM/DD/YYYY

What was the main reason for your most recent emergency department or Urgent Care visit (you can look at the papers you received at discharge from the hospital)?

- Suspected COVID-19 infection
- Asthma
- Chronic obstructive pulmonary disease
- Pneumonia
- Common flu
- Heart attack
- Arrhythmias
- Other

Please specify the main reason for your most recent emergency department or Urgent Care visit.

Click here to finish

Sections

Monthly Surveys

Mood Survey

Monthly Surveys

Over the last 2 weeks, how often have you been bothered by any of the following problems? Tap next to continue.

Little interest or pleasure in doing things.

- Not at all
- Several days
- More than half the days
- Nearly every day

Feeling down, depressed, or hopeless.

- Not at all
- Several days
- More than half the days
- Nearly every day

Trouble falling or staying asleep, or sleeping too much.

- Not at all
- Several days
- More than half the days
- Nearly every day

Feeling tired or having little energy.

- Not at all
- Several days
- More than half the days
- Nearly every day

Poor appetite or overeating.

Not at all

Several days

More than half the days

Nearly every day

Feeling bad about yourself - or that you are a failure or have let yourself or your family down.

Not at all

Several days

More than half the days

Nearly every day

Trouble concentrating on things, such as reading the newspaper or watching television.

Not at all

Several days

More than half the days

Nearly every day

Moving or speaking so slowly that other people could have noticed. Or the opposite - being so fidgety or restless that you have been moving around a lot more than usual.

Not at all

Several days

More than half the days

Nearly every day

Click here to finish

Sections

Monthly Surveys

Anxiety Survey

Monthly Surveys

Becoming easily annoyed or irritable.

- Not at all
- Several days
- More than half the days
- Nearly every day

Over the last two weeks, how often have you been bothered by the following problems? Tap next to continue.

Feeling nervous, anxious, or on edge.

- Not at all
- Several days
- More than half the days
- Nearly every day

Not being able to stop or control worrying.

- Not at all
- Several days
- More than half the days
- Nearly every day

Worrying too much about different things.

- Not at all
- Several days
- More than half the days
- Nearly every day

Trouble relaxing.

- Not at all
- Several days

More than half the days
Nearly every day

Being so restless that it is hard to sit still.

Not at all
Several days
More than half the days
Nearly every day

Feeling afraid as if something awful might happen.

Not at all
Several days
More than half the days
Nearly every day

Click here to finish
