# Supplementary material for: The COVID-19 Citizen Science Study: Protocol for a Longitudinal Digital Health Cohort Study
Source: JMIR Res Protoc. 2021 Aug 30;10(8):e28169. doi: 10.2196/28169 (PMC8407439; doi:10.2196/28169)
Supplement: Multimedia Appendix 2 [file resprot_v10i8e28169_app2.doc]

**Multimedia Appendix 2**. HealthKit Data Types in the COVID-19 Citizen Science Study

| **Category** | **Data Element** |
| --- | --- |
| Activity | Active Energy |
| Cycle Tracking | Basal Body Temperature |
| Nutrition | Biotin |
| Other Data | Blood Alcohol Content |
| Vitals | Blood Glucose |
| Body Measurements | Body Fat Percentage |
| Body Measurements | Body Mass Index |
| Vitals | Body Temperature |
| Nutrition | Caffeine |
| Nutrition | Calcium |
| Nutrition | Carbohydrates |
| Cycle Tracking | Cervical Mucus Quality |
| Nutrition | Chloride |
| Nutrition | Chromium |
| Nutrition | Copper |
| Activity | Cycling Distance |
| Heart | Diastolic Blood Pressure |
| Nutrition | Dietary Cholesterol |
| Nutrition | Dietary Energy |
| Nutrition | Dietary Sugar |
| Body Measurements | Electrodermal Activity |
| Activity | Exercise Minutes |
| Nutrition | Fiber |
| Activity | Flights Climbed |
| Nutrition | Folate |
| Respiratory | Forced Expiratory Volume |
| Respiratory | Forced Vital Capacity |
| Heart | Heart Rate |
| Heart | Heart Rate Variability |
| Body Measurements | Height |
| Respiratory | Inhaler Usage |
| Nutrition | Iodine |
| Nutrition | Iron |
| Body Measurements | Lean Body Mass |
| Nutrition | Magnesium |
| Nutrition | Manganese |
| Cycle Tracking | Menstruation |
| Nutrition | Molybdenum |
| Nutrition | Monounsaturated Fat |
| Nutrition | Niacin |
| Activity | NikeFuel |
| Other Data | Number of Times Fallen |
| Cycle Tracking | Ovulation Test Result |
| Respiratory | Oxygen Saturation |
| Nutrition | Pantothenic Acid |
| Respiratory | Peak Expiratory Flow Rate |
| Heart | Peripheral Perfusion Index |
| Nutrition | Phosphorus |
| Nutrition | Polyunsaturated Fat |
| Nutrition | Potassium |
| Nutrition | Protein |
| Vitals | Respiratory Rate |
| Activity | Resting Energy |
| Heart | Resting Heart Rate |
| Nutrition | Riboflavin |
| Nutrition | Saturated Fat |
| Nutrition | Selenium |
| Cycle Tracking | Sexual Activity |
| Sleep | Sleep |
| Nutrition | Sodium |
| Cycle Tracking | Spotting |
| Activity | Stand Hours |
| Activity | Steps |
| Heart | Systolic Blood Pressure |
| Nutrition | Thiamin |
| Nutrition | Total Fat |
| Other Data | UV Index |
| Nutrition | Vitamin A |
| Nutrition | Vitamin B12 |
| Nutrition | Vitamin B6 |
| Nutrition | Vitamin C |
| Nutrition | Vitamin D |
| Nutrition | Vitamin E |
| Nutrition | Vitamin K |
| Activity | Walking + Running Distance |
| Heart | Walking Heart Rate Average |
| Nutrition | Water |
| Body Measurements | Weight |
| Activity | Workouts |
| Nutrition | Zinc |
