# Supplementary material for: The COVID-19 Citizen Science Study: Protocol for a Longitudinal Digital Health Cohort Study
Source: JMIR Res Protoc. 2021 Aug 30;10(8):e28169. doi: 10.2196/28169 (PMC8407439; doi:10.2196/28169)
Supplement: Multimedia Appendix 3 [file resprot_v10i8e28169_app3.doc]

| Country | N | % |
| --- | --- | --- |
| Afghanistan | 2 | 0.0 |
| Akrotiri | 2 | 0.0 |
| Albania | 3 | 0.0 |
| Algeria | 1 | 0.0 |
| American Samoa | 1 | 0.0 |
| Argentina | 14 | 0.0 |
| Armenia | 1 | 0.0 |
| Australia | 151 | 0.3 |
| Austria | 13 | 0.0 |
| Bahrain | 3 | 0.0 |
| Bangladesh | 6 | 0.0 |
| Barbados | 1 | 0.0 |
| Belarus | 1 | 0.0 |
| Belgium | 34 | 0.1 |
| Bermuda | 2 | 0.0 |
| Brazil | 78 | 0.2 |
| Bulgaria | 2 | 0.0 |
| Burma | 9 | 0.0 |
| Burundi | 1 | 0.0 |
| Cambodia | 1 | 0.0 |
| Cameroon | 1 | 0.0 |
| Canada | 454 | 1.0 |
| Cayman Islands | 1 | 0.0 |
| Chile | 17 | 0.0 |
| China | 7 | 0.0 |
| Colombia | 18 | 0.0 |
| Costa Rica | 5 | 0.0 |
| Croatia | 4 | 0.0 |
| Cuba | 1 | 0.0 |
| Czech Republic | 15 | 0.0 |
| Denmark | 18 | 0.0 |
| Dominican Republic | 8 | 0.0 |
| Ecuador | 8 | 0.0 |
| Egypt | 3 | 0.0 |
| El Salvador | 4 | 0.0 |
| Estonia | 6 | 0.0 |
| Finland | 9 | 0.0 |
| France | 78 | 0.2 |
| Georgia | 4 | 0.0 |
| Germany | 134 | 0.3 |
| Ghana | 1 | 0.0 |
| Greece | 18 | 0.0 |
| Guam | 2 | 0.0 |
| Guatemala | 4 | 0.0 |
| Guernsey | 1 | 0.0 |
| Honduras | 2 | 0.0 |
| Hong Kong | 11 | 0.0 |
| Hungary | 13 | 0.0 |
| Iceland | 4 | 0.0 |
| India | 85 | 0.2 |
| Indonesia | 10 | 0.0 |
| Iran | 8 | 0.0 |
| Iraq | 1 | 0.0 |
| Ireland | 38 | 0.1 |
| Israel | 11 | 0.0 |
| Italy | 110 | 0.2 |
| Jamaica | 2 | 0.0 |
| Japan | 22 | 0.1 |
| Jersey | 1 | 0.0 |
| Jordan | 1 | 0.0 |
| Kenya | 15 | 0.0 |
| Korea South | 2 | 0.0 |
| Kuwait | 1 | 0.0 |
| Kyrgyzstan | 1 | 0.0 |
| Latvia | 3 | 0.0 |
| Lebanon | 2 | 0.0 |
| Lesotho | 1 | 0.0 |
| Lithuania | 3 | 0.0 |
| Luxembourg | 2 | 0.0 |
| Macedonia | 2 | 0.0 |
| Malaysia | 12 | 0.0 |
| Malta | 3 | 0.0 |
| Mexico | 75 | 0.2 |
| Moldova | 1 | 0.0 |
| Morocco | 3 | 0.0 |
| Namibia | 1 | 0.0 |
| Nepal | 1 | 0.0 |
| Netherlands | 99 | 0.2 |
| Netherlands | 1 | 0.0 |
| New Zealand | 24 | 0.1 |
| Nicaragua | 1 | 0.0 |
| Nigeria | 21 | 0.0 |
| Norway | 21 | 0.0 |
| Pakistan | 5 | 0.0 |
| Panama | 4 | 0.0 |
| Paraguay | 2 | 0.0 |
| Peru | 8 | 0.0 |
| Philippines | 115 | 0.3 |
| Poland | 20 | 0.0 |
| Portugal | 35 | 0.1 |
| Puerto Rico | 28 | 0.1 |
| Qatar | 3 | 0.0 |
| Romania | 37 | 0.1 |
| Russia | 25 | 0.1 |
| Saudi Arabia | 6 | 0.0 |
| Serbia and Montenegro | 3 | 0.0 |
| Singapore | 23 | 0.1 |
| Slovakia | 8 | 0.0 |
| Slovenia | 5 | 0.0 |
| South Africa | 77 | 0.2 |
| Spain | 62 | 0.1 |
| Sri Lanka | 1 | 0.0 |
| Sweden | 115 | 0.3 |
| Switzerland | 25 | 0.1 |
| Syria | 1 | 0.0 |
| Taiwan | 8 | 0.0 |
| Tanzania | 3 | 0.0 |
| Thailand | 8 | 0.0 |
| Tunisia | 1 | 0.0 |
| Turkey | 6 | 0.0 |
| Uganda | 2 | 0.0 |
| Ukraine | 8 | 0.0 |
| United Arab Emirates | 12 | 0.0 |
| United Kingdom | 423 | 1.0 |
| United States | 41058 | 93.5 |
| Uruguay | 1 | 0.0 |
| Venezuela | 4 | 0.0 |
| Vietnam | 2 | 0.0 |
| Country not reported | 2201 |  |
